# Biomechanical effects of offset placement of dental implants in the edentulous posterior mandible

**DOI:** 10.1186/s40729-016-0050-6

**Published:** 2016-06-17

**Authors:** Yuta Shimura, Yuji Sato, Noboru Kitagawa, Miyuki Omori

**Affiliations:** Department of Geriatric Dentistry, Showa University, 2-1-1 Kitasenzoku, Ota-ku, Tokyo 145-8515 Japan

**Keywords:** Offset placement, Three-dimensional finite element analysis, FEA, Amount of compressed displacement, Stress distribution, Strain gauge

## Abstract

**Background:**

Proper implant placement is very important for long-term implant stability. Recently, numerous biomechanical studies have been conducted to clarify the relationship between implant placement and peri-implant stress. The placement of multiple implants in the edentulous posterior mandible has been studied by geometric analysis, three-dimensional finite element analysis (FEA), model experimentation, etc. Offset placement is a technique that reduces peri-implant load. However, few studies have used multiple analyses to clarify the value of the offset placement under identical conditions.

The present study aimed to clarify the biomechanical effects of offset placement on the peri-implant bone in edentulous posterior mandibles by comparative investigation using FEA and model experimentation with strain gauges.

**Methods:**

Three implants were embedded in an artificial mandible in the parts corresponding to the first premolar, the second premolar, and the first molar. A titanium superstructure was mounted to prepare models (experimental models). Three load points (buccal, central, and lingual) were established on the part of the superstructure corresponding to the first molar. Three types of experimental models, each with a different implant placement, were prepared. In one model, the implants were placed in a straight line; in the other two, the implants in the parts corresponding to the second premolar and the first molar were offset each by a 1-mm increment to the buccal or lingual side. Four strain gauges were applied to the peri-implant bone corresponding to the first molar.

The experimental models were imaged by micro-computed tomography (CT), and FEA models were constructed from the CT data. A vertical load of 100 N was applied on the three load points in the experimental models and in the FEA models. The extent of compressed displacement and the strain in the peri-implant bone were compared between the experimental models and the FEA models.

**Results:**

Both experimental and FEA models suffered the least compressed displacement during central loading in all placements. The greatest stress and compressive strain was on the load side in all types of placements.

**Conclusions:**

Offset placement may not necessarily be more biomechanically effective than straight placement in edentulous posterior mandibles.

## Background

Bone remodeling to maintain osseointegration between the bone and implant is absolutely essential to ensure favorable results and long-term stability in implant treatment [1, 2]. Bone remodeling requires that various stresses generated around the bone caused by the occlusal load applied to the implant be within an appropriate range. The concentration of stress at the bone-implant interface, caused by overloading, has been reported to result in bone resorption [3–5]. The stress generated in the implant will vary depending on the placement and the nature of the loading of the implants. Gunne et al. [6] stated that the allocation of the occlusal load was affected by the placement of the implant and the geometric form of the prosthetic device. When implants are being embedded, in contrast to the placement of three implants in a straight line, the term “offset placement” is used for a technique in which the central implant is shifted to the side. Rangert et al. [7] reported that in a patient missing three molars, straight placement of the three implants reduced the load to 67 % of that present when two implants were embedded to make a bridge configuration, while offset placement reduced the load to 33 %. However, they did not discuss the specific method they used to calculate the distribution of the load.

The usefulness of offset placement has been studied by geometric analysis, photoelasticity testing, strain gauging, finite element analysis (FEA), and several other techniques [8–19]. However, each type of analysis has its own disadvantages [19], and few studies have been carried out under ideal conditions. Therefore, it is necessary to evaluate the usefulness of offset placement objectively, by using multiple analyses, so as to eliminate the disadvantages of the individual analyses.

Hence, the purpose of the present study was to clarify the biomechanical effects, such as reduction in strain and displacement of implant, of offset placement on the peri-implant bone in edentulous posterior mandibles, by comparative investigation using FEA and model experimentation with strain gauges.

## Methods

### Fabrication of the experimental model

#### Artificial mandibular bone

**Fig. 1:**
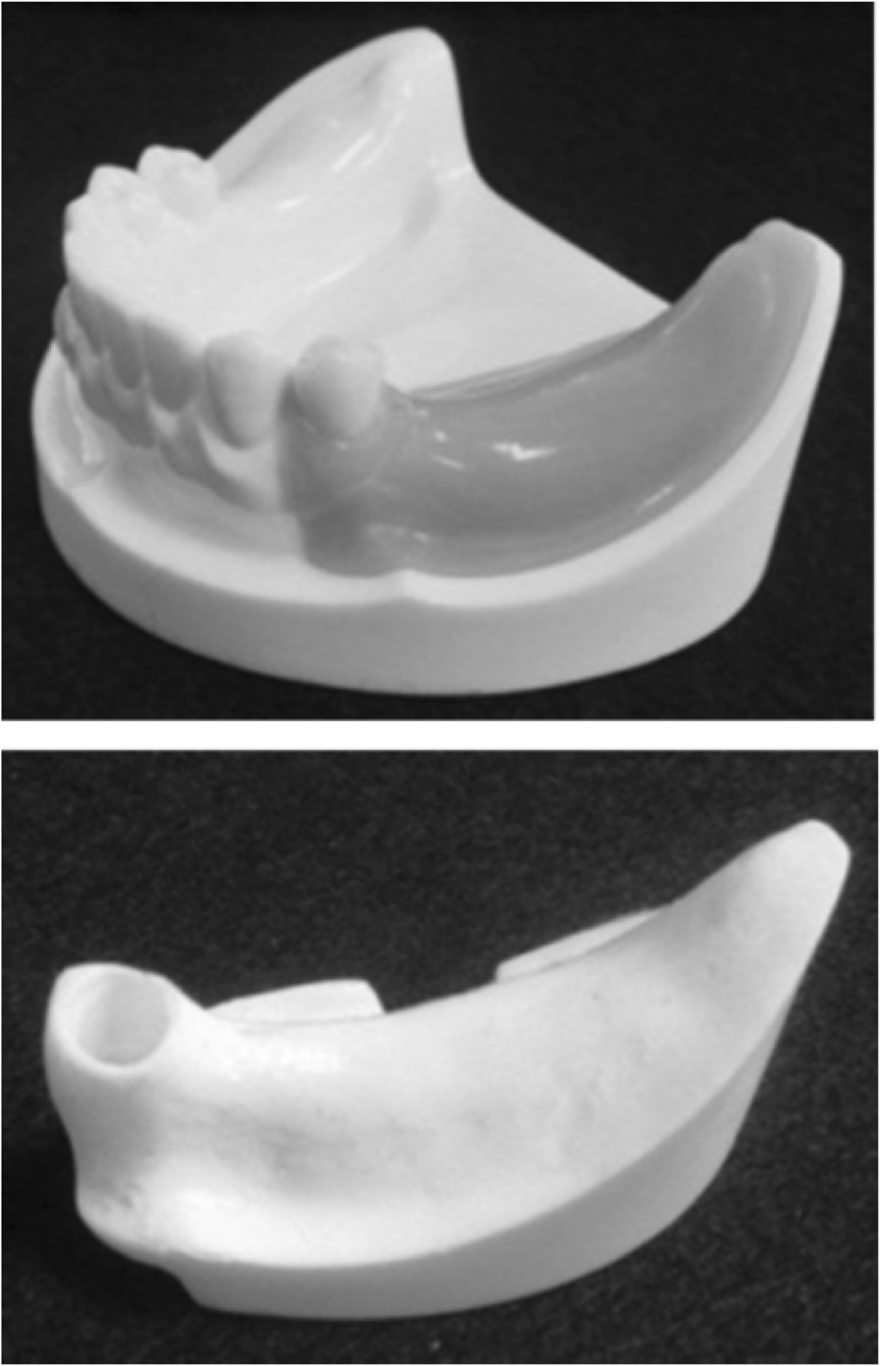
An artificial mandible

An artificial mandibular bone (P9-X.1135, Nissin Dental Products, Kyoto, Japan) with free-end edentulism of the left mandibular first premolar (no. 34), second premolar (no. 35), and first molar (no. 36) was used (Fig. 1). The model was composed of a two-layer structure of artificial cortical bone (urethane resin) and artificial cancellous bone (urethane resin foam).

#### Implant placement

Using the anatomical crown width diameter as a reference [20], we embedded three implants. The distance between the second premolar and mandibular first premolar implants was 8 mm. The distance between the first molar and second premolar implants was 10 mm.

**Fig. 2:**
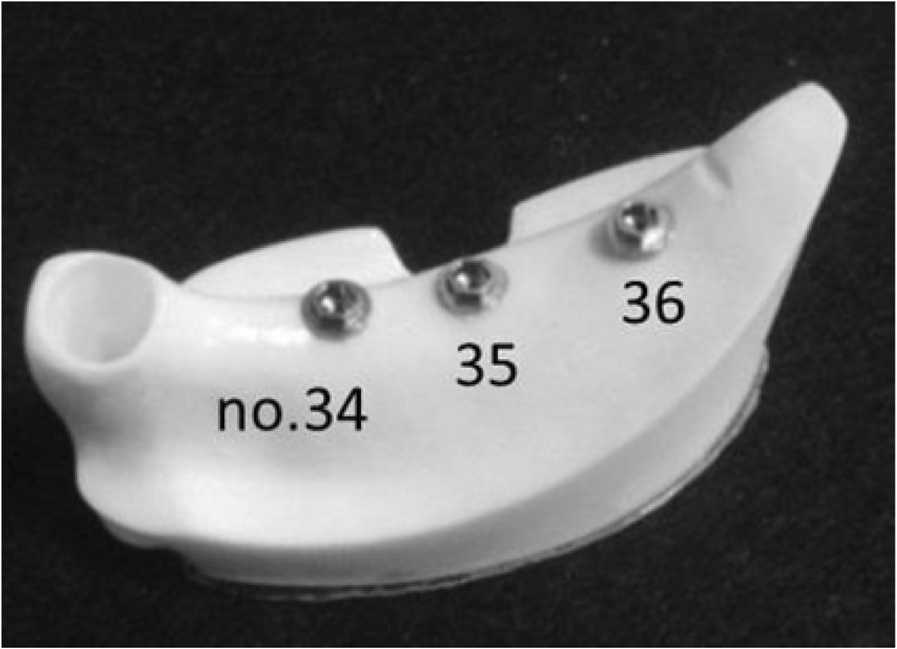
Three implants were embedded in an artificial mandible

An implant placement guide (Landmark Guide™, iCAT, Osaka, Japan) was fabricated to precisely embed the implants in the artificial mandible. A drilling machine (Enkoh’s, Enshu Industrial, Shizuoka, Japan) and implant placement guide were used to embed the implants perpendicular to the bottom surface of the artificial mandible. A drill used to form implant cavities (Brånemark System® Twist Drills, Nobel Biocare, Göteborg, Sweden) was mounted onto the drilling machine, and three implant cavities 3.0 mm in diameter and 10 mm in depth were formed. Then, in each of the implant cavities, an implant 3.75 mm in diameter and 10 mm in length (Brånemark System® Mk III, Nobel Biocare, Göteborg, Sweden) was embedded using 40 N cm of torque (Fig. 2).

**Fig. 3:**
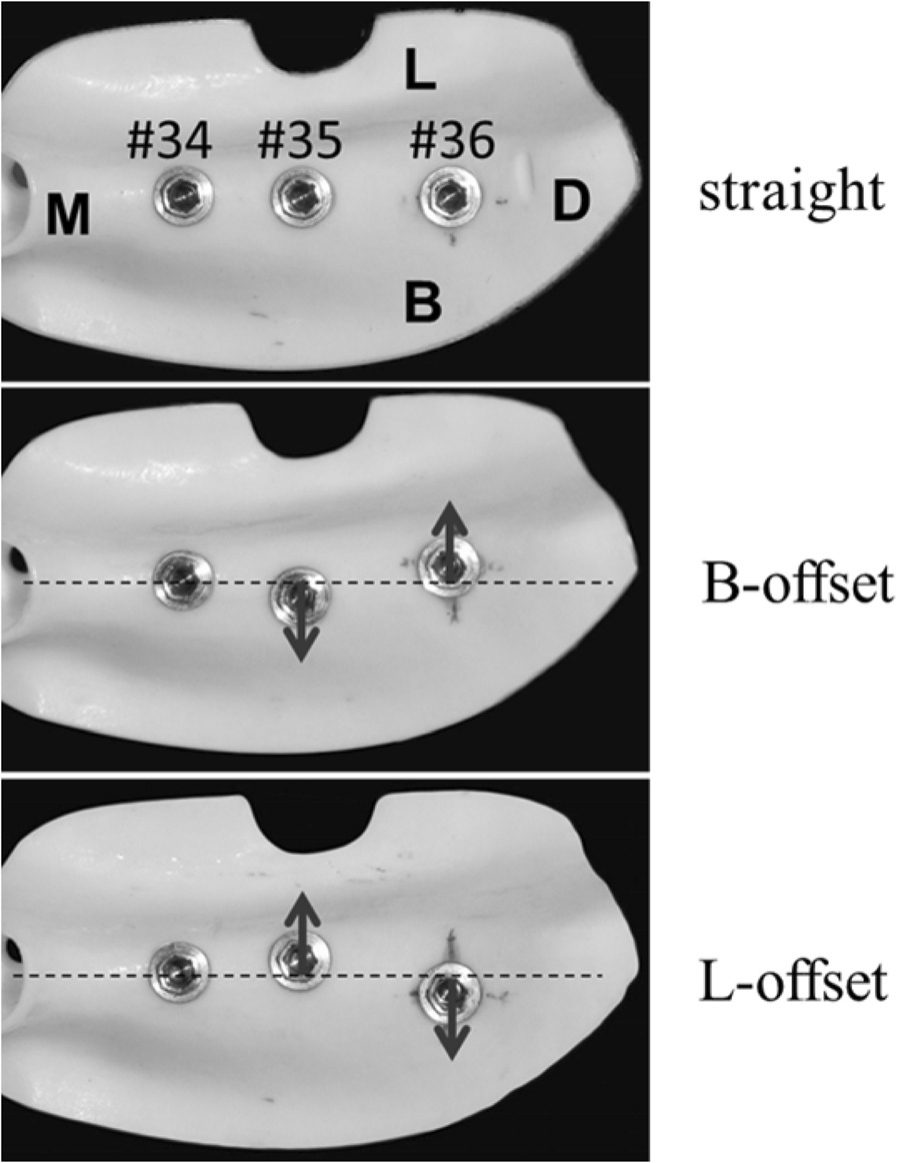
Three different models with different placements

Using models in which three implants were placed in a straight line (straight placement) as a reference, we also prepared two other types of models: (1) no. 35 was offset by 1.0 mm to the buccal side and no. 36 by 1.0 mm to the lingual side (buccal offset placement; B-offset), and (2) no. 35 was offset by 1.0 mm to the lingual side and no. 36 by 1.0 mm to the buccal side (lingual offset placement; L-offset) (Fig. 3). Three experimental models were prepared for each type of placement, i.e., a total of nine experimental models.

#### Preparation of the superstructure

**Fig. 4:**
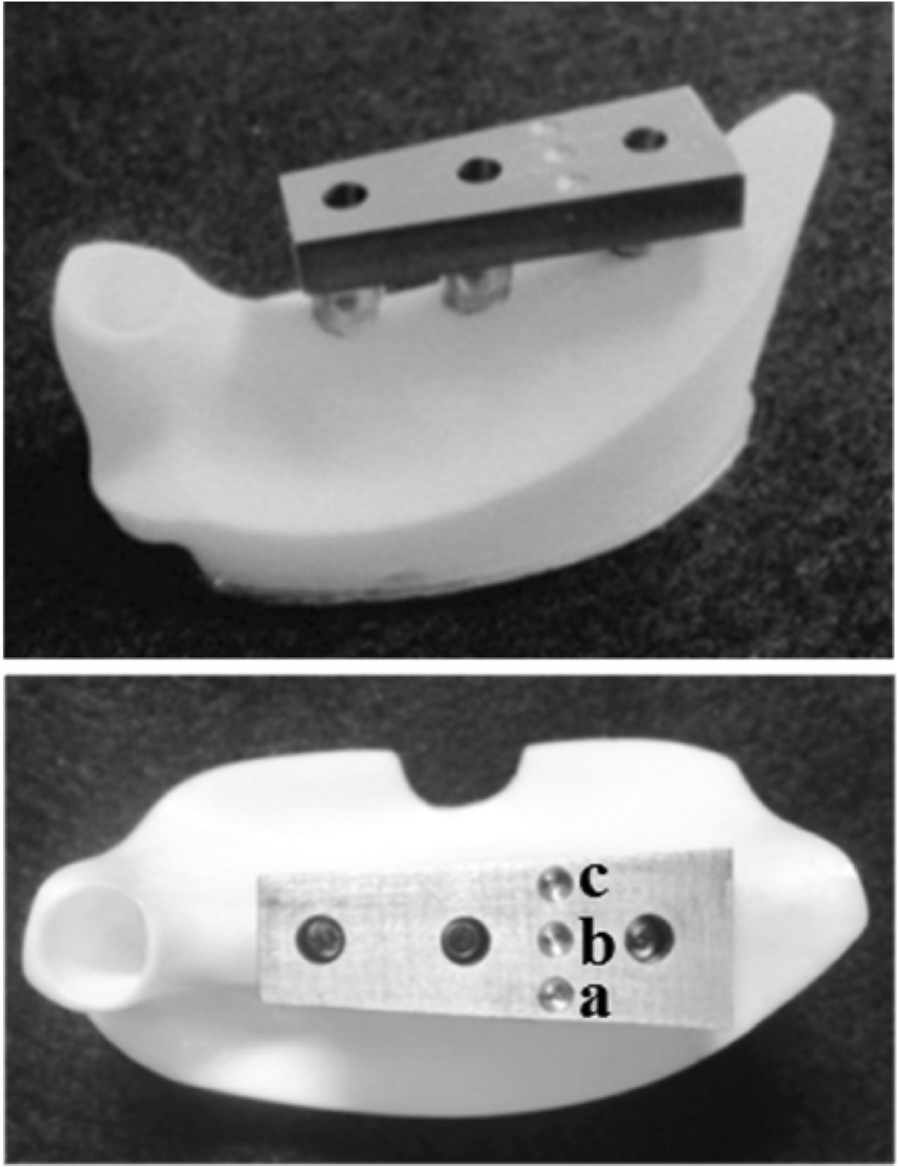
Experimental model. (*a*) Buccal load, (*b*) central load, and (*c*) lingual load

Using the anatomical crown width as a reference [20], it was determined that the occlusal surface view of the superstructure would be trapezoidal with a 7-mm buccolingual width in the mesial first premolar section, a 10-mm buccolingual width in the distal first molar section, and a 26-mm mesiodistal width (Fig. 4). The vertical dimension was 8 mm; the upper 4 mm was the thickness of the superstructure and the lower 4 mm was the abutment connection. Three loading points 2 mm in diameter and 0.2 mm in depth were applied to the occlusal surface of the first molar; these formed the buccal loading point (Fig. 4(a)), central loading point (Fig. 4(b)), and lingual loading point (Fig. 4(c)). The superstructure was made of titanium (ISUS, DENTSPLY Sankin, Tokyo, Japan) and fabricated using computer-aided design/computer-aided manufacturing (CAD/CAM). For each type of placement, we prepared three models by mounting the superstructure onto an artificial mandible model in which implants had been embedded; these served as the experimental models.

#### Application of strain gauges

**Fig. 5:**
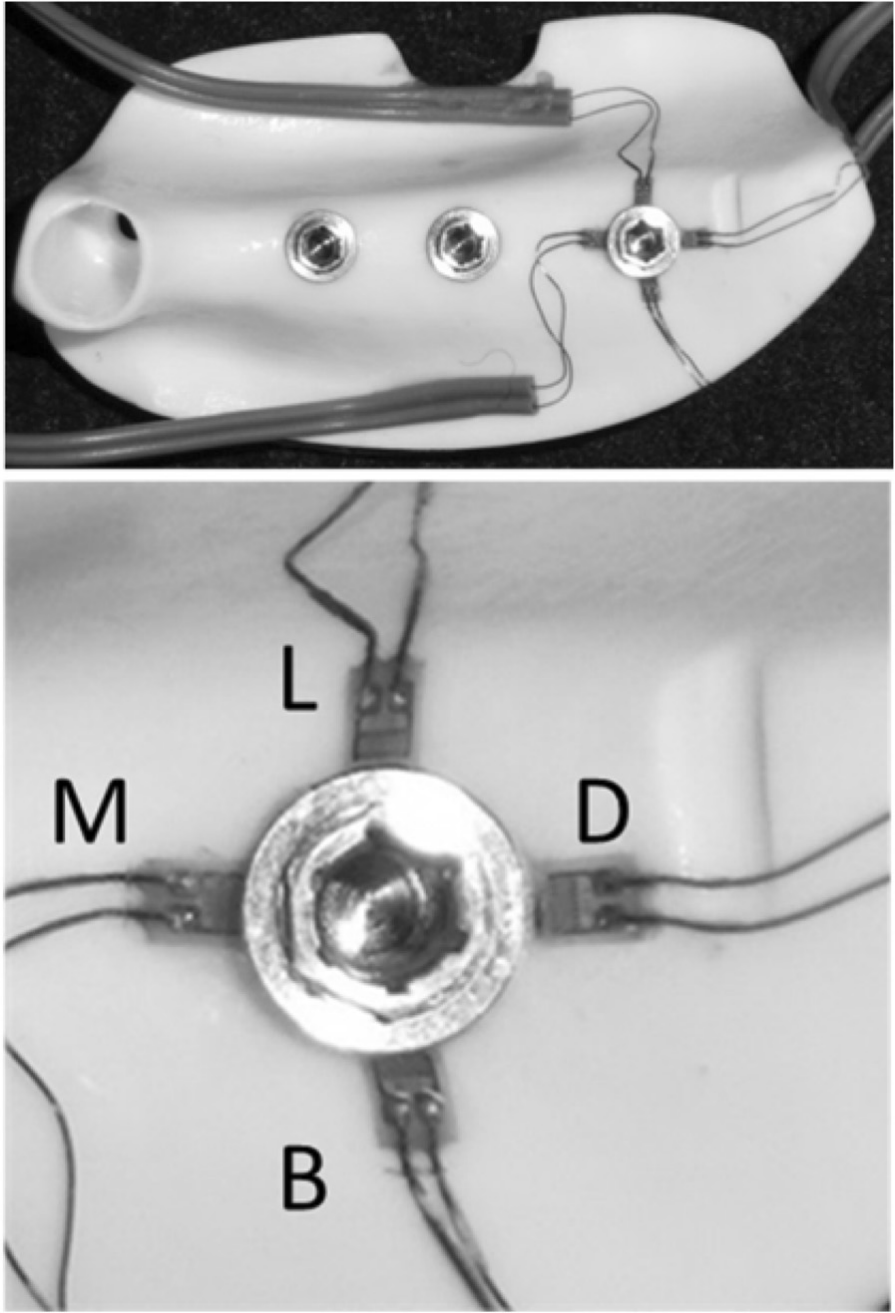
Application of strain gauges

Four two-wire strain gauges (KFR-02N-120-C1-11, Kyowa Electronic Instruments, Tokyo, Japan) were applied to the no. 36 peri-implant bone surface [21]. The surface of the measurement site was polished with no. 320 sandpaper and then wiped clean with acetone, following which they were adhered with a special adhesive (CC-33A, Kyowa Electronic Instruments, Tokyo, Japan). The strain gauges were applied at four places—the mesial, distal, buccal, and lingual sides of the implant—and the strains they measured were designated as strain M, D, B, and L, respectively (Fig. 5).

### Construction of three-dimensional FEA models

The experimental models were fixed in a micro-CT scanner (ScanXmate-L090H, Comscantecno, Kanagawa, Japan) and scanned under the following imaging conditions: tube voltage, 90 kV; tube current, 10 μA; and slice thickness, 106 μm. FEA software (Mechanical Finder®, Research Center of Computational Mechanics, Tokyo, Japan) was used to construct three-dimensional FEA models from the resulting CT data. The mesh was constructed of tetrahedral elements, and the total numbers of nodes and elements were approximately 260,000 and 1,400,000, respectively. FEA models were prepared with appropriate physical properties (Table 1) determined by consulting the values published by the manufacturer of the artificial mandible models and Young’s modulus and Poisson’s ratio used in past research [22]. The implant, abutment, and superstructure were assumed to be a continuous structure made of titanium; no intervening conditions were set between the implant and abutment nor between the abutment and superstructure. The artificial cortical bone, artificial cancellous bone, implant, and superstructure were assumed to be homogeneous, isotropic, and linearly elastic. The boundary conditions for the implant and bone were a state of contact. The coefficient of friction of the interface between the implants and artificial mandibular bones was set to zero. The boundary conditions of the experimental model were reproduced by the contact model of FEA. Immediate loading was assumed in this model, because a state of contact was reproduced between the implant and artificial mandibular bone. The FEA models were made so as to correspond to each of the three experimental models with the respective placements, so nine FEA models were prepared similar to the experimental models.

**Table 1:** Mechanical properties of materials used in the FEA models

Material	Young’s modulus (MPa)	Poisson’s ratio
Artificial cancellous bone	6.29	0.3
Artificial cortical bone	13.73	0.3
Implant and superstructure	108,000	0.3

*FEA* finite element analysis

### Displacement measurements

#### Implant displacement measurements under loading conditions in the experimental model

**Fig. 6:**
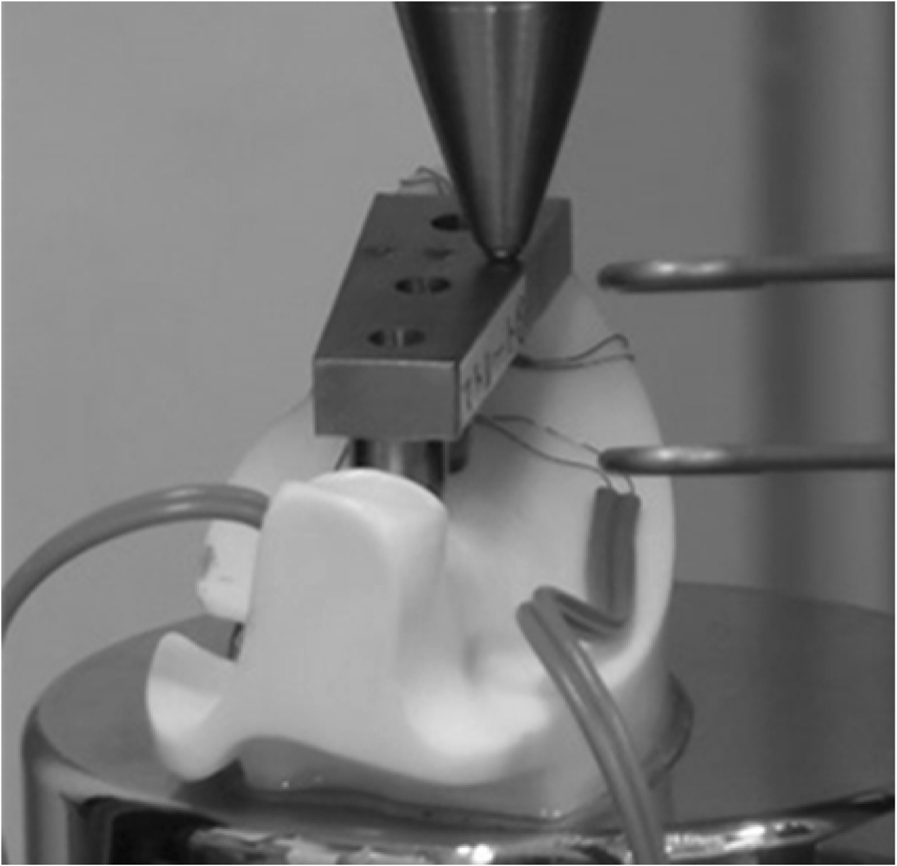
Loading test in the experimental model

Implant displacement under loading conditions was measured using an Instron-type universal testing machine (Instron-5500R®, Instron Japan, Kanagawa, Japan) for the experimental model. The experimental models were placed on the worktable of an Instron-type universal testing machine, and compression tests were performed using a conical jig. A vertical load was applied at a rate of 0.5 mm/s on the three loading points. Using a report [23] stating that the maximum occlusal force applied to an implant superstructure in the molar region is 200 N as a reference, we selected 100 N for loading, to simulate the forces generated during mastication. A strain gauge (2630-100, Instron Japan, Kanagawa, Japan) was attached between the worktable and jig, and the change in the distance between the worktable and jig was measured under the assumption that it would be the same as the implant displacements under loading conditions (Fig. 6). Measurements were taken five times at each loading site, and the mean of the five measurements was considered the representative value of the loading site in that model.

#### Implant displacement measurements under loading conditions in the FEA models

**Fig. 7:**
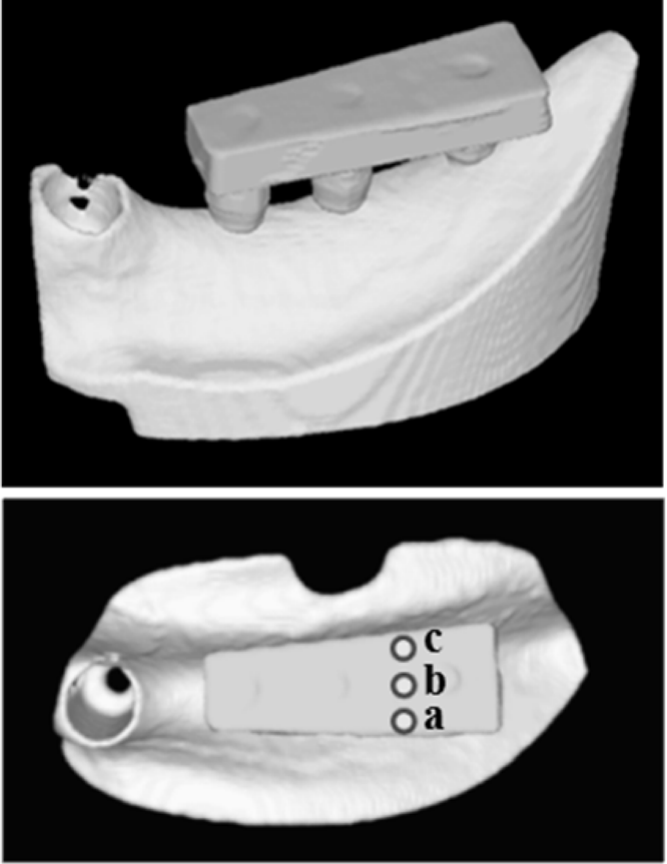
A finite element analysis (FEA) model. (*a*) Buccal load, (*b*) central load, and (*c*) lingual load

All nodes at the bottom of the artificial mandible were completely restrained, 100 N of vertical load was applied to the three loading points, and an elastic analysis was performed. The vertical displacement of the loading points was assumed to be the displacement of the implants under loading conditions, and analyses were performed for the three loading sites (Fig. 7).

#### Measurements of the three-dimensional displacement in the FEA models

We analyzed the three-dimensional displacements of the three implants when 100 N of vertical load was applied. The assessment sites were the neck and tip of the implant, and the displacements of the implants under loading were analyzed with respect to the buccolingual direction (*x*-axis), the mesiodistal direction (*y*-axis), and the inferior-superior direction (*z*-axis).

### Measurement of strain

#### Measurement of strain in the experimental models

An Instron-type universal testing machine was used to run a compression test during which strain was measured simultaneously. The strain gauges and a laptop computer (Latitude E5500, Dell, Texas, USA) were connected to a sensor interface (PCD-300B, Kyowa Electronic Instruments, Tokyo, Japan), and the strain in the peri-implant bone during the application of a 100-N load was measured.

#### Measurement of strain in the FEA models

The places on the experimental models where the strain gauges were applied were represented as coordinate points on the FEA models, and the strain in the FEA models was calculated by dividing the change in length between before and after loading by the length of the strain gauges.

### Assessment of the stress distribution in the FEA models

An equivalent stress occurring in the peri-implant bone during loading was observed and assessed in a buccolingual cross-section of the no. 36 implant. The stress distributions were compared between placements.

### Statistical analysis

Compressed displacement and strain values were examined by two-way analysis of variance using differences in load site and placement as factors. The level of significance was set to 5 %. Subsequently, Tukey’s method was used to perform a multiple comparison test, and for this also, the level of significance was set to 5 %.

PASW Statistics Ver18 (SPSS, Tokyo, Japan) was used for statistical processing.

## Results

### Compressed displacement

**Fig. 8:**
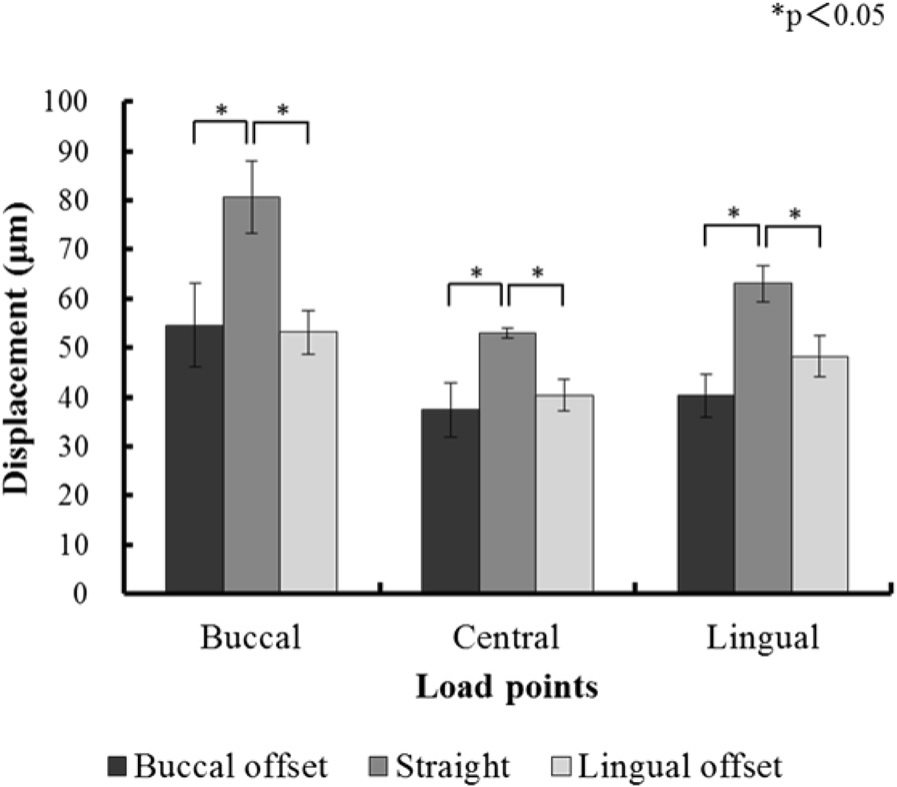
The displacement of the implants under loading in experimental models

**Fig. 9:**
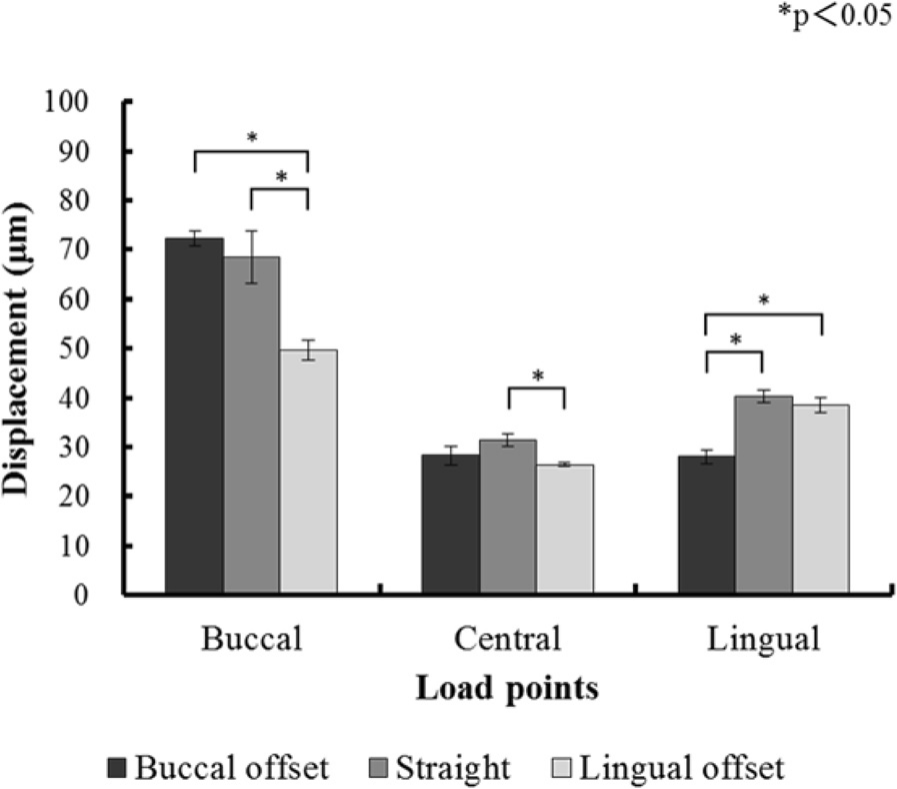
The displacement of the implants under loading in finite element analysis (FEA) models

Figures 8 and 9 and Tables 2 and 3 show the results for the compressed displacement of the implants, by loading site, during the application of a 100-N vertical load in each of the models.

**Table 2:** Means and standard deviations (SD) of displacement of the implants (μm) under loading in experimental models

Models	Displacement (μm)		
	Buccal loading	Central loading	Lingual loading
Buccal offset	54.60 ± 8.53	37.39 ± 5.53	40.22 ± 4.24
Straight	80.66 ± 7.47	52.92 ± 1.07	63.03 ± 3.69
Lingual offset	53.11 ± 4.35	40.36 ± 3.18	48.22 ± 4.18

**Table 3:** Means and standard deviations (SD) of displacement of the implants (μm) under loading in finite element analysis (FEA) models

Models	Displacement (μm)		
	Buccal loading	Central loading	Lingual loading
Buccal offset	72.24 ± 1.43	28.24 ± 1.86	28.02 ± 1.41
Straight	68.49 ± 5.24	31.43 ± 1.23	40.18 ± 1.29
Lingual offset	49.63 ± 2.03	26.39 ± 0.37	38.44 ± 1.46

In all placements, the compressed displacement in the experimental models and FEA models was greatest with buccal loading and smallest with central loading at the three loading points. For both the experimental models and the FEA models, the compressed displacement during buccal loading with straight placement was significantly greater than that with the L-offset (*P* < 0.05). The compressed displacement during lingual loading with straight placement was significantly greater than that with the B-offset (*P* < 0.05).

### Three-dimensional displacement in the FEA models

**Fig. 10:**
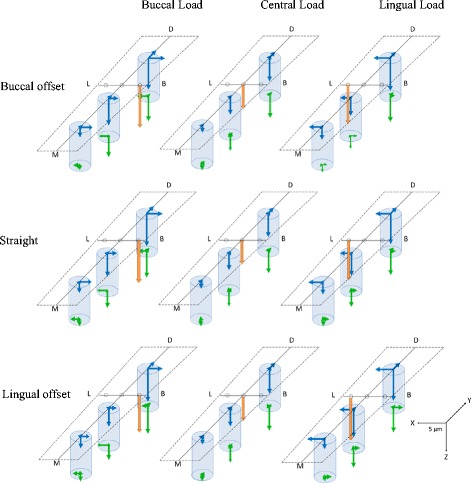
The displacement of the three implants

Figure 10 shows the results for the three-dimensional displacement of the implants, by loading site, during the application of a 100-N vertical load. Displacement in the buccolingual direction (*x*-axis direction) during buccal loading exhibited movement such that the implant neck was displaced to the buccal side, the implant tip was displaced to the lingual side, and the implant body rotated and tilted to the loading side. There was little displacement of the implant tip during lingual loading, while the implant body showed movement with the displacement to the lingual side. The least displacement was observed during central loading. Displacement in the mesiodistal direction (*y*-axis direction) was such that the three implant bodies were rotated and tilted distally at all three loading sites. The three-dimensional displacement in the vertical direction (*z*-axis direction) was such that in all three loading sites, no. 36 was displaced the most, and the more mesial the implant body, the lesser the displacement was, and the distal parts sank. The least displacement was during central loading; buccal loading and lingual loading exhibited similar displacements. Between placements, similar trends were found, and differences in implant placement were not found to affect the three-dimensional displacement or the direction of displacement.

### Strain on the peri-implant bone

#### Strain in the experimental models

**Fig. 11:**
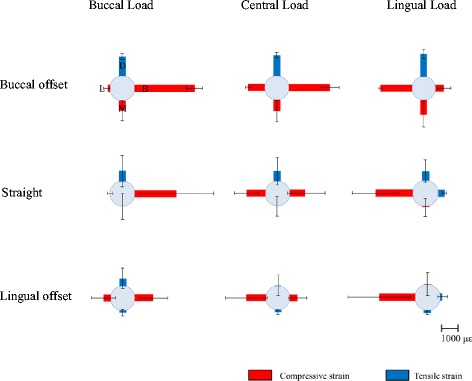
The strain around the no. 36 implant in the experimental models

Figure 11 and Table 4 show the strain, by loading site, in the implant part corresponding to the first molar in the experimental models during the application of a 100-N vertical load.

**Table 4:** Means and standard deviations (SD) of strain around the no. 36 implant (με) in the experimental models

Models	Loading	Strain (με)			
		Strain M	Strain B	Strain D	Strain L
Buccal offset	Buccal loading	−599.33 ± 595.46	−4507.35 ± 1192.62	1164.77 ± 169.94	−148.09 ± 174.19
	Central loading	−697.29 ± 651.92	−2526.69 ± 570.91	1272.93 ± 144.11	−1038.21 ± 151.61
	Lingual loading	−928.19 ± 779.29	−536.22 ± 431.65	1458.01 ± 281.15	−1997.40 ± 93.43
Straight	Buccal loading	−0.64 ± 762.69	−2462.40 ± 2202.81	650.61 ± 926.87	7.84 ± 171.37
	Central loading	7.80 ± 597.37	−907.59 ± 1126.39	608.12 ± 804.44	−1131.15 ± 713.48
	Lingual loading	−46.63 ± 503.19	374.18 ± 85.59	535.71 ± 624.55	−2135.81 ± 1341.85
Lingual offset	Buccal loading	137.71 ± 198.50	−1116.87 ± 846.02	442.81 ± 620.37	−441.78 ± 735.38
	Central loading	157.83 ± 137.02	−494.15 ± 565.61	18.56 ± 645.98	−1236.11 ± 1290.83
	Lingual loading	171.26 ± 104.51	125.42 ± 283.74	−234.42 ± 662.75	−2085.35 ± 1804.50

Considerable compressive strain was observed with the load-side strain gauges in all placements, and similar trends were observed between placements. As much as about 4500 με of compressive strain was observed. Strain B was significantly higher for the B-offset than for any of the other placements during buccal loading (Table 5). Strain L did not show a significant difference between placements during lingual loading (Table 6).

**Table 5:** Tukey’s test for strain B in the experimental models

Models		Mean difference	*P* value
Straight	B-offset	1524.82	0.044
Straight	L-offset	−503.40	0.670
B-offset	L-offset	−2028.22	0.007

**Table 6:** Tukey’s test for strain L in the experimental models

Models		Mean difference	*P* value
Straight	B-offset	−25.14	0.999
Straight	L-offset	168.04	0.948
B-offset	L-offset	193.18	0.932

#### Strain in the FEA models

**Fig. 12:**
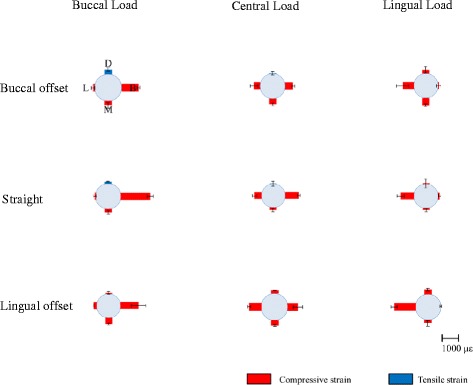
The strain around the no. 36 implant in the finite element analysis (FEA) models

Figure 12 and Table 7 show the strain, by loading site, in the implant part corresponding to the first molar in the FEA models during the application of a 100-N vertical load.

**Table 7:** Means and standard deviations (SD) of strain around the no. 36 implant (με) in the finite element analysis (FEA) models

Models	Loading	Strain (με)			
		Strain M	Strain B	Strain D	Strain L
Buccal offset	Buccal loading	−222.34 ± 158.56	−934.84 ± 76.82	252.11 ± 103.72	−98.32 95.94
	Central loading	−336.26 ± 94.27	−477.17 ± 87.68	35.05 ± 102.98	−385.89 ± 240.13
	Lingual loading	−448.13 ± 78.51	−49.85 ± 139.33	−210.45 ± 206.82	−676.08 ± 383.54
Straight	Buccal loading	−203.99 ± 99.82	−1717.66 ± 179.51	124.50 ± 30.18	−72.26 ± 86.36
	Central loading	−151.55 ± 110.05	−857.84 ± 83.22	21.12 ± 157.01	−447.40 ± 140.88
	Lingual loading	−94.09 ± 97.43	−95.19 ± 81.18	−47.47 ± 275.05	−817.47 ± 215.71
Lingual offset	Buccal loading	−397.70 ± 51.11	−1121.79 ± 453.39	−68.01 ± 87.44	−204.14 ± 14.05
	Central loading	−293.21 ± 56.29	−574.07 ± 261.34	−176.54 ± 16.66	−696.83 ± 87.11
	Lingual loading	−175.14 ± 171.13	−3.47 ± 51.47	−289.54 ± 78.95	−1248.16 ± 180.57

Similar to the experimental models, considerable compressive strain was observed on the load side in all placements, and similar trends were noted between placements. As much as about 1700 με of compressive strain was observed. Strain B was significantly greater with straight placement than with offset placement during buccal loading (Table 8). Stain L with lingual loading was significantly higher in the L-offset than in the B-offset (Table 9). There were differences between the experimental and FEA models in the magnitude of strain around the peri-implant bone. However, the trends for the occurrence of strain based on differences in loading site were similar between the two groups.

**Table 8:** Tukey’s test for strain B in the FEA models

Models		Mean difference	*P* value
Straight	B-offset	−402.94	0.007
Straight	L-offset	−323.79	0.029
B-offset	L-offset	79.16	0.772

**Table 9:** Tukey’s test for strain L in the FEA models

Models		Mean difference	*P* value
Straight	B-offset	−58.94	0.855
Straight	L-offset	270.67	0.061
B-offset	L-offset	329.61	0.020

### Stress distributions in the FEA models

**Fig. 13:**
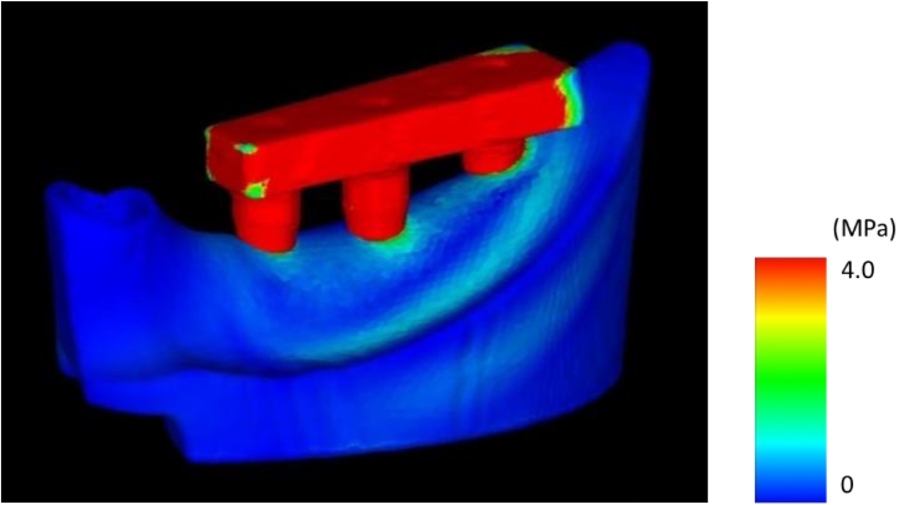
The distribution of equivalent stress around the peri-implant bone in the finite element analysis (FEA) models

**Fig. 14:**
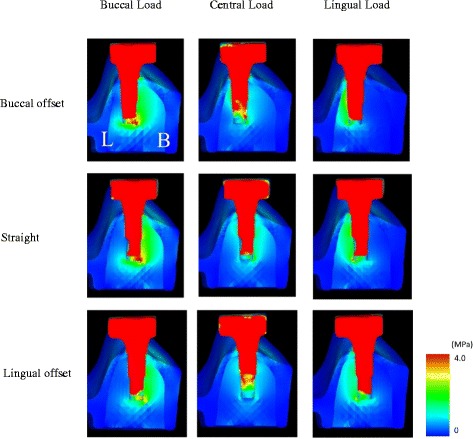
The distribution of equivalent stress around the no. 36 implant in the finite element analysis (FEA) models

Figures 13 and 14 show the stress distributions in the peri-implant bone in the FEA models during the application of a 100-N vertical load. In all placements, considerable stress was concentrated in the load-side peri-implant bone. Stress was found to be concentrated over a broad range in the cancellous bone surrounding the implant body bottom in the B-offset during buccal loading and in the L-offset during lingual loading.

## Discussion

### Experimental models

Reported studies verifying the effects of offset placement include ones where implant bodies were embedded in rectangular experimental models [11–14], ones where rectangular bone models were constructed with FEA models [15, 16], and ones where FEA models were constructed from CT data on human mandibles [17, 18]. The artificial mandible models used in the present study were type II in the Lekholm and Zarb classification [24] and created using clinically valid estimates of bone quality, bone structure, and bone morphology. If the effects of implant placement alone are being verified, then models that are simple in form can be used. However, artificial mandibles were used in the present study in order to take into account the constraints on offset amounts due to anatomical morphology, the differences in stress distribution in the surrounding bone due to bone morphology, and other aspects of clinical practice.

### FEA models

Analysis was done with FEA models, in which the cancellous bone was simplified as being a homogeneous body. FEA models have been reported to be useful for verifying the behavior of implants when loaded [22], and so, the effects of different placements were verified with FEA models and compared with the results from the experimental models. In a previous study, Omori et al. compared the compressed displacement between experimental models and FEA models to verify the validity of the FEA models [22]. They reported that the constraint conditions in the artificial mandible bottom and differences in the actual Young’s modulus were two reasons why the two models produced different absolute values for the compressed displacement. In order to apply the actual Young’s modulus to the FEA models in the present study, FEA models with a known Young’s modulus were created and the resulting values of compressed displacement were compared with the results from the experimental models; after the appropriate Young’s modulus was found, the FEA models were created again for further analysis. In previous studies verifying the usefulness of offset placement, one set of FEA models was created and analyzed by changing the conditions or settings. Few studies used different FEA models with the same placement models [15–17]. In the present study, we carried out the same experiments with both the models to verify the validity of each analysis.

Moreover, considering the possibility of error while using an implant placement guide, we created many FEA models for each placement to compare the accuracy between the same placement models used with different FEA models.

### Use of multiple analyses

Most studies verifying the usefulness of offset placement used a single technique for analysis [8–19]. Therefore, the results regarding the usefulness of offset placement vary depending on the techniques used in the studies, and hence, the issue remains controversial. The present study aimed to evaluate the usefulness of offset placement objectively by using two analyses—experimental and FEA models—and to verify the validity of each analysis. The limitation with experimental models is that it is not possible to observe internal stress, while FEA models have the disadvantage that it is required to confirm the agreement of the results with those obtained from the oral cavity of the living body. In the present study, we believe that it was possible to evaluate the usefulness of offset placement objectively by using multiple analyses so that the disadvantage of each analysis was compensated for by the other.

### Sites of strain measurement

There is reportedly a concentration of stress in the surrounding cortical bone when occlusal load is applied to an implant [22, 25, 26]. Based on this, the strain gauges were applied to the peri-implant bone surface in the present study as well.

### Experimental results

#### Amount of compressed displacement

**Fig. 15:**
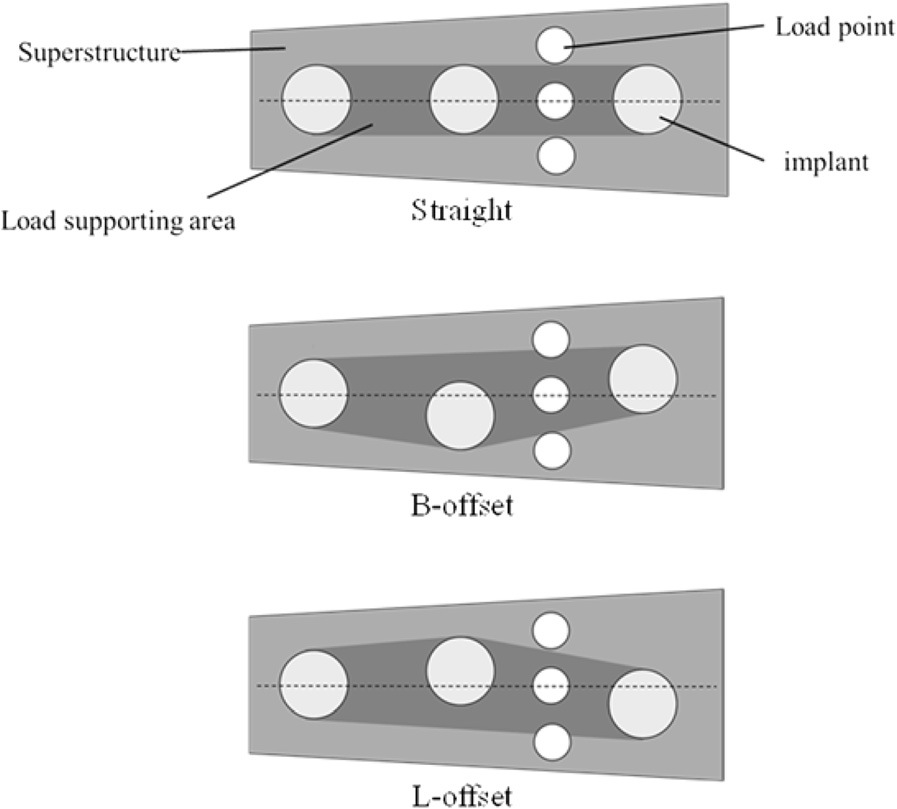
Load supporting area in the superstructures

The experimental models and FEA models were similar and exhibited the same trends for compressed displacement based on differences in loading sites. In all placements, central loading resulted in the least compressed displacement and buccal loading the greatest. Thus, compressed displacement exhibited the same trend in the experimental models and FEA models. The results of both models may be reliable. When the effects of offset placement are considered, there is the concept of the load-supporting area (Fig. 15) put forth by Sato [27]. The load-supporting area is the area surrounded by the lines connecting the implant peripheries, and if a loading point falls within this area, there is little lateral force on the implant body. In the L-offset with buccal loading and the B-offset with lingual loading conditions, where the loading points are close to the load-supporting area, there is significantly less force than there is with straight placement (*P* < 0.05). Compared to the experimental models, the FEA models showed 10 μm less compressed displacement, but this may be the influence of the minute perturbations caused by differences in the constraint conditions in the artificial mandible bottom. The ability to completely reproduce the behaviors of experimental models with the FEA models would produce results with better approximation.

#### Three-dimensional displacement in the FEA models

Similar trends were observed in the direction and magnitude of displacement between placements. Buccal loading exhibited considerable motion towards the buccal rotation/tilting of the implant bodies, and lingual loading exhibited little motion towards lingual displacement. This corresponds to the fact that there was more compressed displacement during buccal loading than during lingual loading.

#### Strain on the peri-implant bone

In past reports where models were used, it was reported that compressive strain in the peri-implant bone occurs in sites close to the loading side and tensile strain occurs on the side opposite to the loading side [25, 28]. Concentration of stress leads to bone resorption [3–5]. In the experimental models in the present study as well, considerable compressive strain was observed in the loading-side peri-implant bone. Strain in the FEA models also exhibited considerable compressive strain on the loading side, similar to the experimental models. In terms of quantitative data for comparison with the experimental models, the length of the places where the strain gauges were applied was measured on the FEA models and the strain was calculated from the length before and after loading and compared with the experimental models. In previous verifications of offset placement with FEA models [15–18], the maximum stress that occurs in the peri-implant bone has been measured and compared with the observed stress distribution with straight placement. In the present study, we measured strain at the same sites in the experimental models and the FEA models, and therefore, we believe that it was possible to carry out a more multifaceted and objective assessment of the mechanical effects on the surrounding bone. The strain values for the experimental and FEA models showed similar trends with change in the loading site and placement of implants. However, the values of strain were different between the experimental and FEA models, the difference being greater than 10-fold at several sites. While comparing the experimental and FEA models, the difference in the accuracy of the cancellous bone and the difference in boundary conditions for the implant and the bone would affect the strain values. The cancellous bone in the experimental model was made of urethane resin foam that mimicked the trabecular structure. The cancellous bone of the FEA model, on the other hand, had a uniform structure, not a trabecular one. This seems to be the reason behind the difference in the strain values between the experimental and FEA models. While determining the boundary conditions between the implant and the artificial mandibular bone, immediate loading was assumed, but not osseointegration. The implant and artificial mandible in the FEA model were in complete contact. The coefficient of friction of the interface between the implants and artificial mandibular bones was set to zero. The boundary conditions of the experimental model were not bonded together, but they were completely fitted together mechanically. The difference in boundary conditions would affect the difference in strain values between the experimental and FEA models. Regarding how different placements affect strain, there was a trend for the strain to be greater when the loading site was at a greater distance from the load-supporting area (Fig. 15). In addition, there was not a significantly less strain site by offset placement. Anitua et al. [29] have reported that offset placement did not affect marginal bone loss around the implant in the oral cavity of the living body. Overloading of the peri-implant bone has been reported to result in bone resorption [3–5], and the concentration of considerable stress in the load-side peri-implant bone observed in our study confirms this. Hence, we conclude that our observation that offset placement did not reduce the stress around the peri-implant bone in both the experimental and FEA models is similar to that of the clinical report by Anitua et al. In addition, we also conclude that our results confirm the validity of both analyses. Thus, offset placement may not necessarily be more biomechanically effective than straight placement.

#### Stress distribution

Concentration of stress in the loading-side peri-implant bone was observed in all placements and for both the experimental and the FEA models. Considerable stress was also found to be concentrated in the no. 36 peri-implant bone in buccal loading with buccal offset and lingual loading with lingual offset. Similar to the strain results, stress was observed in a large range under conditions where the loading site was far from the load-supporting area (Fig. 15).

### Limitations of the study

This study does have a few limitations. The only items assessed in these experiments were the compressed displacement of the implant bodies and strain in the peri-implant bone. More specifically, the stress and strain applied to the implant bodies themselves, among other items, should be verified in order to verify the effects of offset placement. Moreover, the occlusal loading conditions and the jawbone models used in this study were different from those in the body, and hence, future studies addressing these limitations are needed.

## Conclusions

In the present study, which aimed to verify the biomechanical effects of offset placement on peri-implant bone, we created multiple finite element models and models where implants were actually placed. We compared the compressed displacement as well as the strain and stress distribution in the peri-implant bone between both kinds of models, and the results can be summarized as follows:

1. Central loading resulted in the least compressed displacement in all placements in the experimental models as well as the FEA models.
2. In both the experimental models and the FEA models, compressive stress was observed to be concentrated in the loading-side peri-implant bone.
3. The strain and stress was significantly greater under conditions of offset placement where the loading site was far from the load-supporting area.

These results suggest that compared to straight placement, offset placement is not necessarily more biomechanically effective.
